# Ecological Niche Modelling and nDNA Sequencing Support a New, Morphologically Cryptic Beetle Species Unveiled by DNA Barcoding

**DOI:** 10.1371/journal.pone.0016662

**Published:** 2011-02-09

**Authors:** Oliver Hawlitschek, Nick Porch, Lars Hendrich, Michael Balke

**Affiliations:** 1 Department of Entomology, Zoological State Collection, Munich, Germany; 2 School of Life and Environmental Sciences, Deakin University, Melbourne, Australia; 3 GeoBioCenter, Ludwig-Maximilians-University, Munich, Germany; University of Copenhagen, Denmark

## Abstract

**Background:**

DNA sequencing techniques used to estimate biodiversity, such as DNA barcoding, may reveal cryptic species. However, disagreements between barcoding and morphological data have already led to controversy. Species delimitation should therefore not be based on mtDNA alone. Here, we explore the use of nDNA and bioclimatic modelling in a new species of aquatic beetle revealed by mtDNA sequence data.

**Methodology/Principal Findings:**

The aquatic beetle fauna of Australia is characterised by high degrees of endemism, including local radiations such as the genus *Antiporus*. *Antiporus femoralis* was previously considered to exist in two disjunct, but morphologically indistinguishable populations in south-western and south-eastern Australia. We constructed a phylogeny of *Antiporus* and detected a deep split between these populations. Diagnostic characters from the highly variable nuclear protein encoding arginine kinase gene confirmed the presence of two isolated populations. We then used ecological niche modelling to examine the climatic niche characteristics of the two populations. All results support the status of the two populations as distinct species. We describe the south-western species as *Antiporus occidentalis* **sp.n.**

**Conclusion/Significance:**

In addition to nDNA sequence data and extended use of mitochondrial sequences, ecological niche modelling has great potential for delineating morphologically cryptic species.

## Introduction

DNA sequencing is an increasingly popular and important tool for the assessment of global species diversity. At present, the mitochondrial cytochrome c oxidase 1 is a standard marker, and “DNA barcoding” or “barcoding” is the name coined for this approach of DNA-based species identification [1]–[8]. Barcoding is an especially valuable tool for conservation planning, as it provides rapidly releasable quantitative biodiversity data and a glimpse of a level of diversity that extends beyond morphologically delineated entities. Barcoding uses short, standardised sequence segments of the genome, and has proven highly useful when researchers are confronted with high expected species numbers and morphologically cryptic groups ([9]–[12], see also [13]). As argued in Burns et al. [14], there exist cases in which morphologically and ecologically well distinguishable species exhibit only minimal divergence in their barcodes, and species delimitation by barcoding should not depend on arbitrarily chosen levels of divergence. Similarly, it remains unclear how to deal taxonomically with cases in which morphologically identical populations exhibit certain amounts of divergence in the mitochondrial genome [9], [10]. Apparently, the conflict between mtDNA sequence data and morphology requires consideration of other character sources in order to delimit species.

The species concept and the delimitation of species have been a matter of controversy since the early days of systematic biology. Efforts have been made to find a concept which encompasses different approaches to the species problem. DeQueiroz [15] suggested the “Unified Species Concept”, which relies on the single definition of species as “existence as a separately evolving metapopulation lineage”. Traditional species concepts like the biological [16], ecological [17], [18] or genotypic cluster [19] species concepts are “secondary species criteria” or “operational criteria”, meaning that not every single criterion must fit every species, but on the other hand more than one of these criteria may be appropriate to a species. They rather act as tools to delimit species.

Among many other possible characters, ecological factors should help delimit species, assuming that each species has formed its own particular niche. However, even for sister-species pairs having detectably distinct niches, the collection of life history data is usually problematic. Ecological Niche Modelling (ENM) is one possible approach to this problem, using widely available environmental data and universally available georeferenced distributional records as a proxy for species ecology. Van Valen [17] and Andersson [18] argued that species can be understood as groups of individuals occupying the same niche or adaptive zone. Explicit models can be based on species locality data and a raster representing various environmental, mainly climatic, variables (bioclimatic modelling). They have demonstrated their ability in circumscribing species' ecological niches and assessing their potential distributions [20]. Such models always remain restricted to a small selection of environmental variables, but nevertheless have been shown to be capable of predicting potential distributions of species and estimating the impact of the ecological variables studied [21]. Particularly when integrated with phylogenetic studies, ENMs have also proven to be a powerful tool in species delimitation [22]–[27]. To our knowledge no such studies have yet been conducted for beetles, a group for which generally relatively few analyses using ENM approaches exist [28]–[30].

Modelling approaches can aid in species delimitation only if the species studied actually diverge in their response to the environmental variables incorporated in the analysis [31]. Evidence suggests that niche conservatism, i.e., the stability of ecological niches over time, is a common pattern in closely related species and that it is a major force driving allopatric speciation [32]–[37]. Kozak and Wiens [35] postulated that certain North American salamander species are allopatric because of their inability to tolerate the climatic conditions in the lowland areas between their highland habitats, even if these differences appear relatively minor. However, most of these case studies concerned sibling species inhabiting climatically similar areas. Other studies present evidence for niche divergence between sibling species, for distributions of closely related species on environmental axes and for niche divergence as a speciation mechanism [23], [38]–[40]. This apparent contradiction suggests that neither assumption is valid for all groups of organisms and that both cases can occur in closely related species and may contribute to speciation.

We conducted a molecular biodiversity assessment of Australian diving beetles, using 3′ cytochrome c oxidase 1 sequences [41] and found divergence between geographically separated populations of one species, *Antiporus femoralis* (Boheman, 1958). In the absence of morphological differences, we evaluated other data sources and suggest that ecological niche modelling and nDNA characters provide evidence for the presence of a new, cryptic species which we will describe below.

## Materials and Methods

### Study group: Australian diving beetles

Australia's diverse and highly characteristic diving beetle (Dytiscidae) fauna offers many opportunities to study speciation and radiation events. To date, almost 300 dytiscid species are known, of which approximately 90% are endemic to the continent, belonging to 18 or 19 exclusively Australian radiations [42]–[47]. Many endemic species of diving beetle are not widespread, but rather restricted to certain climatic regions, river drainage systems or other geographical features. In southern Australia, the arid Nullarbor Plain with a West-East-extent of more than 1200 kilometres acts as a very potent geographical barrier for freshwater organisms due to its arheic conditions and its virtual lack of surface water [48]. Many groups, including the diving beetles, show patterns of disjunct distributions in south-western and south-eastern Australia, excluding the Nullarbor Plain. Geological evidence shows that this situation is a result of rather recent events [49]–[52]. During the Miocene, vast stretches of southern Australia, including the Nullarbor Plain, were covered by seas during marine intrusions, with lush tropical forests growing in the humid climate along its coast. Only after regression of sea levels, from about 10 to 6.6 million years before present, did the area fall dry and the humid conditions make way for today's arid climate.

The genus *Antiporus* Sharp, 1882 (tribe Hydroporini Aubé, 1836), with 16 described species to date [53]–[57], is distributed in still or slow-flowing water, mainly in south-eastern and south-western Australia, along the east coast of the continent and with one species in the Northern Territory, north-western Australia and northern Queensland. An additional species is distributed widely across New Zealand in different habitats. Watts [54] described two additional species from Western Australia, *A. pembertoni* Watts, 1997 and *A. hollingsworthi* Watts, 1997. Four additional species (*A. mcraeae* Watts and Pinder, 2000, *A. pennifoldae* Watts and Pinder, 2000, *A. gottwaldi* Hendrich, 2001 and *A. kalbarriensis* Hendrich and Watts, 2010) have been described recently. Most *Antiporus* species are restricted to the southwest, to the eastern coast or to south-eastern Australia, and some show remarkable regional endemism. However, two disjunct populations of *A. femoralis* (Boheman, 1958) have been reported from south-western and south-eastern Australia and were considered conspecific because of the lack of morphological differences (e.g. Watts [53], Brancucci [58]). In this study, we focus on these two *A. femoralis* populations.

### DNA sequencing and data analysis

We preserved a part of our collections in pure ethanol in the field and later extracted DNA for sequencing, employing methods explained in detail in Balke et al. [59] and Hendrich and Balke [60].

For a population-level screening of all Australian diving beetles, we sequenced the 3′ end of cytochrome c oxidase 1 (cox1) [41]. In a second step, we sequenced additional genes to infer phylogenetic relations within the present focal clade, *Antiporus*. Genes and primers used for sequencing are given in Table S1. After detection of a possible cryptic species, we sequenced a fragment of the nuclear protein coding gene arginine kinase (ARK).

Sequences were submitted to GenBank and are publicly available under accession numbers FR727264 to FR727325 and as part of a general *cox1* dataset of Australian Dytiscidae (FR 732513 to FR 733591). Individual beetles from which we extracted and sequenced DNA all bear a green cardboard label that indicates the DNA extraction number of M. Balke (e.g. “DNA 2000 M.Balke”). This number links the DNA sample, the dry mounted voucher specimen and GenBank entries.

We ran analyses for two separate datasets. Dataset one included 24 specimens from all available *Antiporus* species and four outgroup taxa, and 2953 characters over all five DNA loci. Dataset 2 included 70 specimens: all available *A. femoralis* specimens (n = 30) and other *Antiporus* species as outgroups. We used 799 characters of cytochrome c oxidase 1 only. The following analyses were all performed on the CIPRES portal 2.2 [61] unless stated otherwise. Both datasets were aligned using the program MUSCLE 3.7 [62]. We used jModeltest 0.1.1 [63] to choose appropriate substitution models.

We ran maximum likelihood analyses using the program GARLI [64] until 10,000 generations revealed no significant improvement of likelihood scores of the topology. We then ran resampling with 250 bootstrap replicates.

We also used Bayesian analyses with the program MrBayes 3.0 [65]. Each of two runs consisted of 4 chains which ran for 1,000,000 generations, with samplefreq = 1,000 and 25% burnin fraction. Convergence between runs and posterior probabilities of the estimates was determined by plotting the log likelihoods in Excel.

Finally, we used parsimony searches to infer phylogenetic relations as implemented in the program TNT version 1.1 (on a local desktop computer), which we also used to run 500 jackknife (removal 36%) replications to assess node stability [66] (hit best tree 5 times, keep 10,000 in memory).

Pairwise distances were computed using the Kimura 2-parameter model in MEGA 4.0 [67].We used the sequence editor Se-Al v2.0a11 (http://tree.bio.ed.ac.uk/software/seal/) to detect diagnostic characters.

### Ecological niche modelling

We used the Maxent 3.3.2 [68] software for modelling the potential distribution of the two major clades of *A. femoralis* detected in the phylogenetic analyses. Maxent follows the Maximum Entropy principle [69] and combines presence-only data and environmental layers to create a gridded model of the potential distribution of the target species. Several studies have shown that Maxent produces better results than comparable methods [70], [71] and have confirmed its ability to predict a species' distribution outside its known range [72]–[75]. It has also been frequently used in phylogeographic studies [76], [77], some having taxonomic implications [24], [27], [78]. We obtained a total of 80 distribution points of *A. femoralis* (61 from eastern Australia and 19 from western Australia, Table S2) from our own databases (Hendrich unpublished) and from the ANIC database (http://anic.ento.csiro.au/database/biota_details.aspx). We excluded a single doubtful New Zealand locality that might refer to *A. uncifer* Sharp, 1882. Climate data was obtained from the worldclim database ([79], http://www.worldclim.org). We used the bioclimatic variables at a resolution of 2.5 arc-minutes. These 19 variables likely summarise dimensions of climate of special importance for determining species distributions [80].

As proposed by several authors [81], [82], inclusion of too many of these climate variables may cause “over-fitting” problems, as many represent similar and highly correlated dimensions of climate. Furthermore, a specific selection of predictors according to natural history properties of the target species may significantly enhance the reliability of ENMs [37]. Rödder and Lötters [83] also showed that transferability of models across space requires careful attention. To avoid misleading results, Environmental variables should be chosen with special care when models are used to predict species' distributions outside their native range.

*A. femoralis* inhabits summer-dry wetlands and rest pools of small rivers and creeks having a high seasonal variation in water volume, many of which fall almost completely dry during the dry season (November to March). Thus, precipitation and its seasonal variation is the climatic factor assumed to have the highest impact on the long-term persistence of *A. femoralis* populations. Temperature may also be important as higher insolation and thus higher temperature causes drought. Therefore, aside from “annual mean temperature” and “annual precipitation”, we chose factors representing the interaction of precipitation and temperature and the seasonality of these factors, i.e., “precipitation warmest quarter”, “precipitation coldest quarter” and “precipitation seasonality”. This latter factor gives a direct measurement of the strength of the seasonality, whereas values of precipitation of the warmest and coolest quarters indicate its direction.

We used the default Maxent settings with a random test percentage of 25% of the input localities set aside for model testing. We chose the logistic output format, displaying suitability values from 0 (unsuitable) to 1 (optimal) [84]. Jackknifing was performed to measure the importance of the variables. Model validation was conducted by calculating the area under the curve (AUC), which reflects the model's ability to distinguish between presence records and random background points [68], [85]. AUC values range from 0.5 for models without any predictive ability to 1.0 for models with perfect predictive ability. According to Swets [86], AUC values >0.9 are considered to have ‘very good’, >0.8 ‘good’ and >0.7 ‘useful’ discrimination abilities.

We performed ENMs using locality data of *A. femoralis* from eastern Australia and of *A. femoralis* from Western Australia, restricting background data to areas likely to be colonizable for the species as recommended by Phillips et al. [87] Therefore, we manually delimited areas encompassing the known localities, separating them from completely arid areas away from the coast (Fig. S1). We also performed an ENM using data from all *A. femoralis* individuals pooled together. All runs were performed with 100 bootstrap repeats. Test localities were randomly selected anew for each repeat, and mean output values were used as final results.

For further statistical analysis of the modelling results, we used the ENMtools software [88]. We measured niche overlap of *A. femoralis* from eastern Australia and of *A. femoralis* from Western Australia using Schoener's D [89] and the I statistic, modified from the Hellinger distance [90].

We also used two hypothesis tests included in ENMtools. First, we used the niche identity test to determine whether the ENMs generated for the two species are identical or exhibit statistically significant difference. The test combines the samples of both species into a common pool. Under the assumption that the species behave interchangeably in their use of ecological niche space, their identities are randomized, and two new samples with the same sizes as the original samples are extracted. By repeating this process, a set of pseudoreplicates is generated. The results are compared with the true calculated niche overlap (see above). The lower the true niche overlap in comparison to the scores created by the pseudoreplicates of the pooled samples is, the more significant the niche difference between the two species compared.

Second, we used the background test to evaluate the null hypothesis that all divergence in the ecological niches of two taxa, given that the niches are represented by two sets of localities, can be explained by the differences in their environmental feature spaces. Specifically, we use it to ascertain whether ENMs of *A. femoralis* from eastern Australia and of *A. femoralis* from western Australia are more or less similar than expected based on the environmental differences in their completely disjunct ranges. This test is particularly appropriate for allopatric species because in many cases, distinct geographic spaces provide a different set of environmental conditions. That is, differences in ENMs may result from niche space availability rather than from niche diversification [30]. The test places random occurrence points within the range of one of the two species to be compared and measures niche similarity between these points and the original localities of the second species. If the true measured overlap values are significantly higher (or lower) than the values generated by the background test, the null hypothesis that ENMs are more similar (or divergent) than based on habitat availability is rejected. This test is conducted in both directions, and different directions may yield different results.We performed the identity test, as well as background tests in both directions, with 500 iterations.

### Morphology and taxonomy

Specimen depositories:

ANICAustralian Insect Collection, Canberra, Australia

CFPCollection Fernando Pederzani, Ravenna, Italy

CLHCollection Lars Hendrich, Berlin, Germany, property of NMW

CSRCollection Saverio Rocchi, Firenze, Italy

NMWNaturhistorisches Museum Wien, Austria

SAMASouth Australian Museum, Adelaide, South Australia, Australia

WAMWestern Australian Museum, Perth, Western Australia, Australia

ZSMZoological State Collection, Munich, Germany

Beetles were examined using a Leica MZ 12.5 dissecting scope at 10–100x. Male genitalia were studied and figured in wet conditions. Images of male genitalia were made using incident light and a digital photo imaging system, composed of a Leica DM 2500 M microscope and a Tucsen 5.0 MP camera. The microscope was fitted with Leica HCX PL “Fluotar” 5x and 10x metallurgical grade lenses [91]. Habitus images were taken with a Nikon D700, equipped with a bellows and Leica Photar 2.8/25 mm lens. Image stacks were aligned and assembled in Helicon Focus 4.77TM.

The terminology to denote the orientation of the genitalia follows Miller and Nilsson [92]. Coordinates are given in decimal notation unless cited verbatim from labels. To determine the position of these localities, we used various Australian road maps and Google Earth (http://earth.google.com).

### Nomenclatural acts

The electronic version of this document does not represent a published work according to the International Code of Zoological Nomenclature (ICZN), and hence, the nomenclatural acts contained in the electronic version are not available under that Code from the electronic edition. Therefore, a separate edition of this document was produced by a method that ensures numerous identical and durable copies, and those copies were simultaneously obtainable (from the publication date noted on the first page of this article) for the purpose of providing a public and permanent scientific record, in accordance with Article 8.1 of the Code. The separate print-only edition is available on request from PLoS by sending a request to PLoS ONE, 185 Berry Street, Suite 3100, San Francisco, CA 94107, USA along with a cheque for US $10 (to cover printing and postage) payable to “Public Library of Science”.

In addition, this published work and the nomenclatural acts it contains have been registered in ZooBank (http://zoobank.org/), the proposed online registration system for the ICZN. The ZooBank LSIDs (Life Science Identifiers) can be resolved, and the associated information can be viewed, through any standard web browser by appending the LSID to the prefix “http://zoobank.org/”. The LSID for this publication is: urn:lsid:zoobank.org:pub:445E5E19-C6A5-46C5-84AF-B35986BB7AAE.

We deposit printed copies of the work in the libraries of:

CSIRO Entomology (Canberra, urn:lsid:biocol.org:col:32981),

Natural History Museum London (urn:lsid:biocol.org:col:1009),

Naturhistorisches Museum Wien (Vienna, urn:lsid:biocol.org:col:34043),

Queensland Museum (Brisbane, urn:lsid:biocol.org:col:34161),

South Australian Museum (Adelaide, urn:lsid:biocol.org:col:34244) and

Zoologische Staatssammlung (Munich, urn:lsid:biocol.org:col:34660).

## Results

### Molecular phylogenetics

jModeltest selected the GTR+G model for all gene regions but cytochrome c oxidase 1 and histone 3, for which the GTR+I+G model was selected. These models were used for all further analyses. Where partitioning was not possible, the GTR+G model was used.

**Figure 1:**
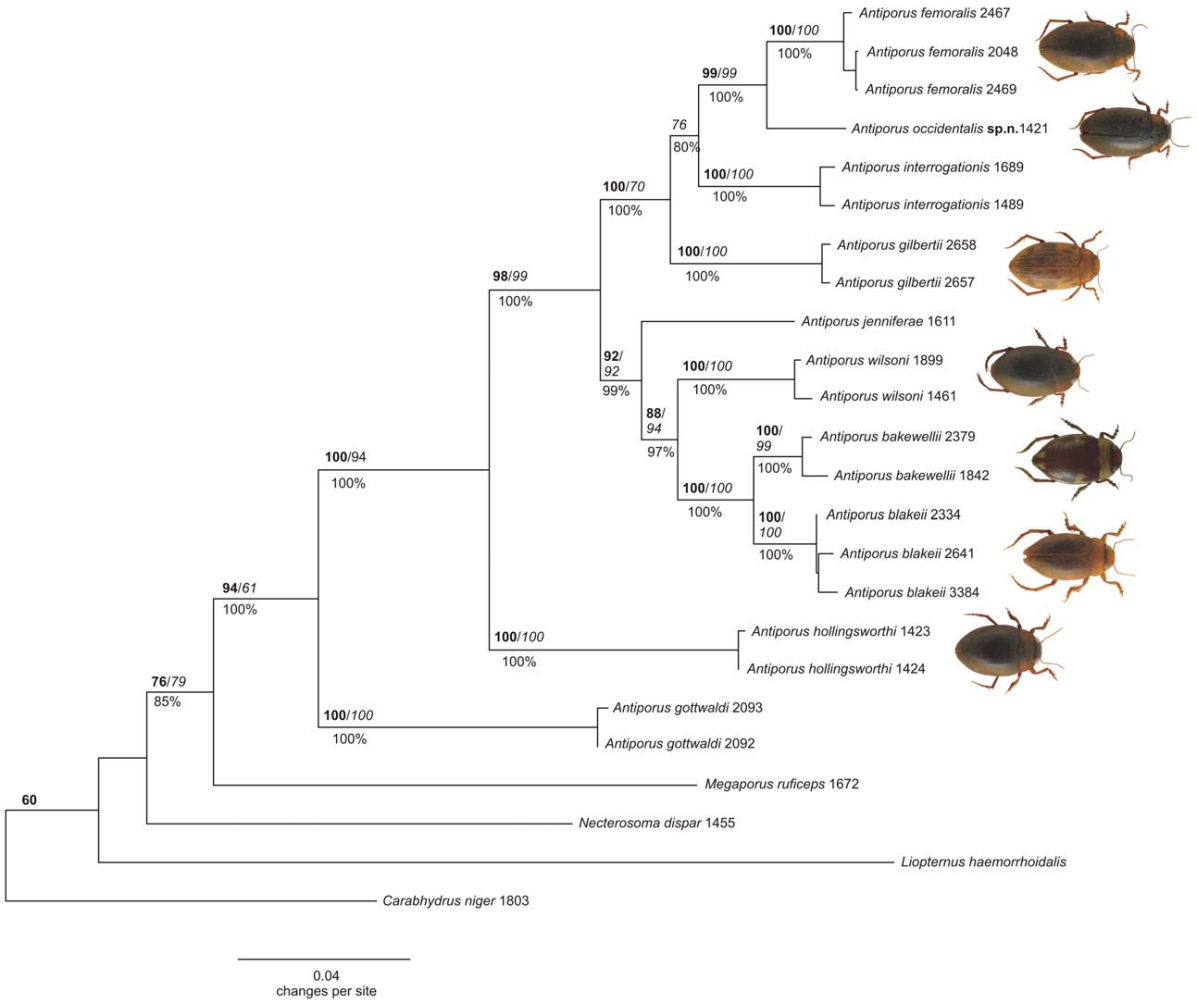
Phylogram of the genus *Antiporus*. The phylogram is based on a maximum likelihood tree with 5 gene loci and 2953 characters made in GARLI. Branch values are: GARLI bootstrap (bold/above branch), TNT jackknife (italic/above branch), and MrBayes posterior probability (below branch). Each tip represents one specimen. Specimen collection numbers are given after the species name.

Maximum likelihood, Bayesian and parsimony analysis of a multigene dataset of Australian *Antiporus* all yielded very similar topologies with generally significant node support values (Fig. 1). Four specimens that we initially identified as *Antiporus femoralis* always formed a monophyletic group, but the single Western Australian specimen diverged from the remaining three specimens, all from the eastern part of Australia, by 6.5%. The sister species of that clade is either *A. interrogationis* or *A. gilbertii*. The Bayesian analysis supported *A. interrogationis* as sister taxon to the *A. femoralis* clade. Maximum likelihood and parsimony analyses yielded a clade comprising *A. interrogationis* and *A. gilbertii* as sister group to the *A. femoralis* clade, albeit with support values of less than 60 in both cases (not shown).

**Figure 2:**
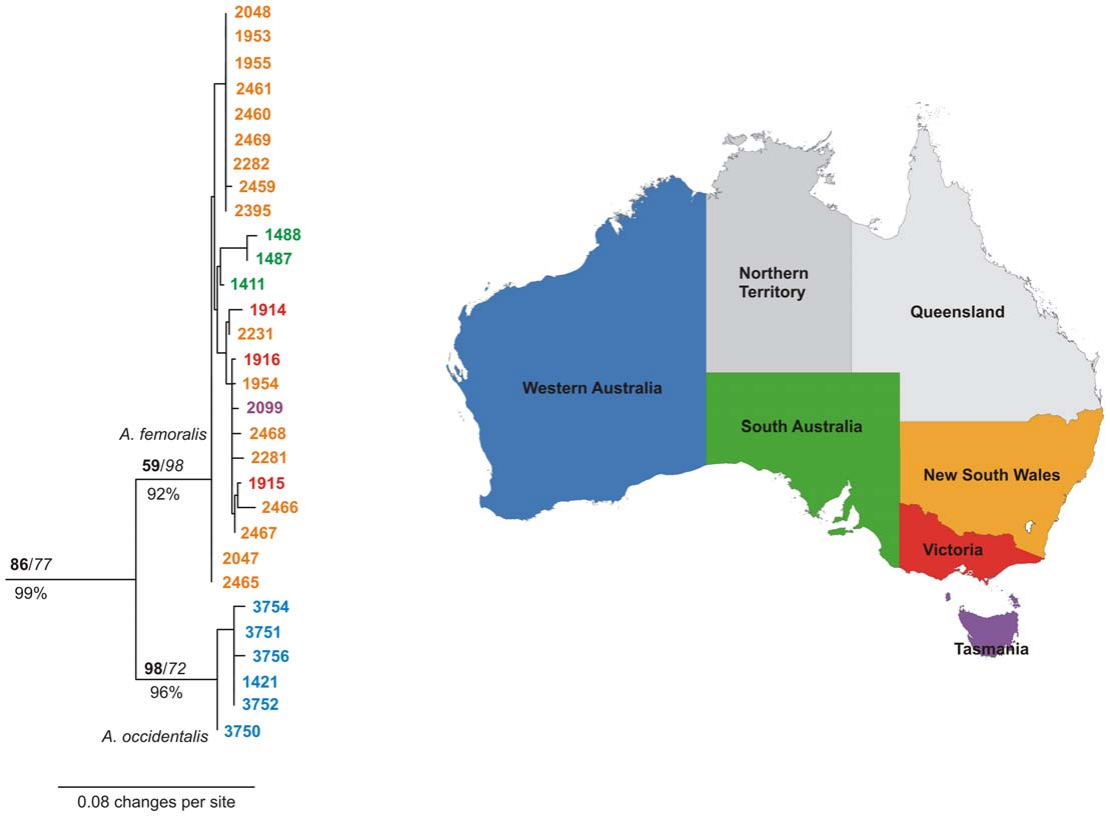
Phylogram of *Antiporus femoralis* and *Antiporus occidentalis* sp.n. Tree based on a cytochrome c oxidase 1 tree with 799 characters made in GARLI. Branch values are: GARLI bootstrap (bold), TNT jackknife (italic), and MrBayes posterior probability (below branch). Each tip represents one specimen. Outgroups (A. *interrogationis*, *A. jenniferae*, *A. wilsoni*, *A. bakewellii*, *A. blakeii*, *A. gilbertii*, *A. hollingsworthi*, *A. gottwaldi* and *Sternopriscus eikei*) are not shown. Colours of specimen numbers represent their state of origin, see map of Australia on the right.

Analysis of cox1 for 30 specimens from the *A. femoralis* clade clearly confirmed a subdivision into a western and an eastern clade (Fig. 2). Within the eastern and western groups of *A. femoralis*, pairwise distances were 0.0% to 2.9% (mean 0.7%±0.7%) for eastern and 0.0% to 1.0% (mean 0.5%±0.3%) for western *A. femoralis*. The divergence between the two clades was 3.5% to 6.6% (mean 4.46%±0.6%).

Within eastern *A. femoralis*, only specimens from South Australia seem to form a monophyletic group, but this clade of three individuals is not significantly supported. The only morphologically divergent specimen, which is larger and darker and originates from Tasmania (“DNA M. Balke 2099”), is nested in a clade comprising specimens from New South Wales and Victoria.

A 510-bp fragment of the nuclear protein coding gene arginine kinase was successfully amplified for specimens from both clades. The sequence divergence was 1.39%, and six parsimony-informative sites were identified.

### Ecological Niche Modelling

**Figure 3:**
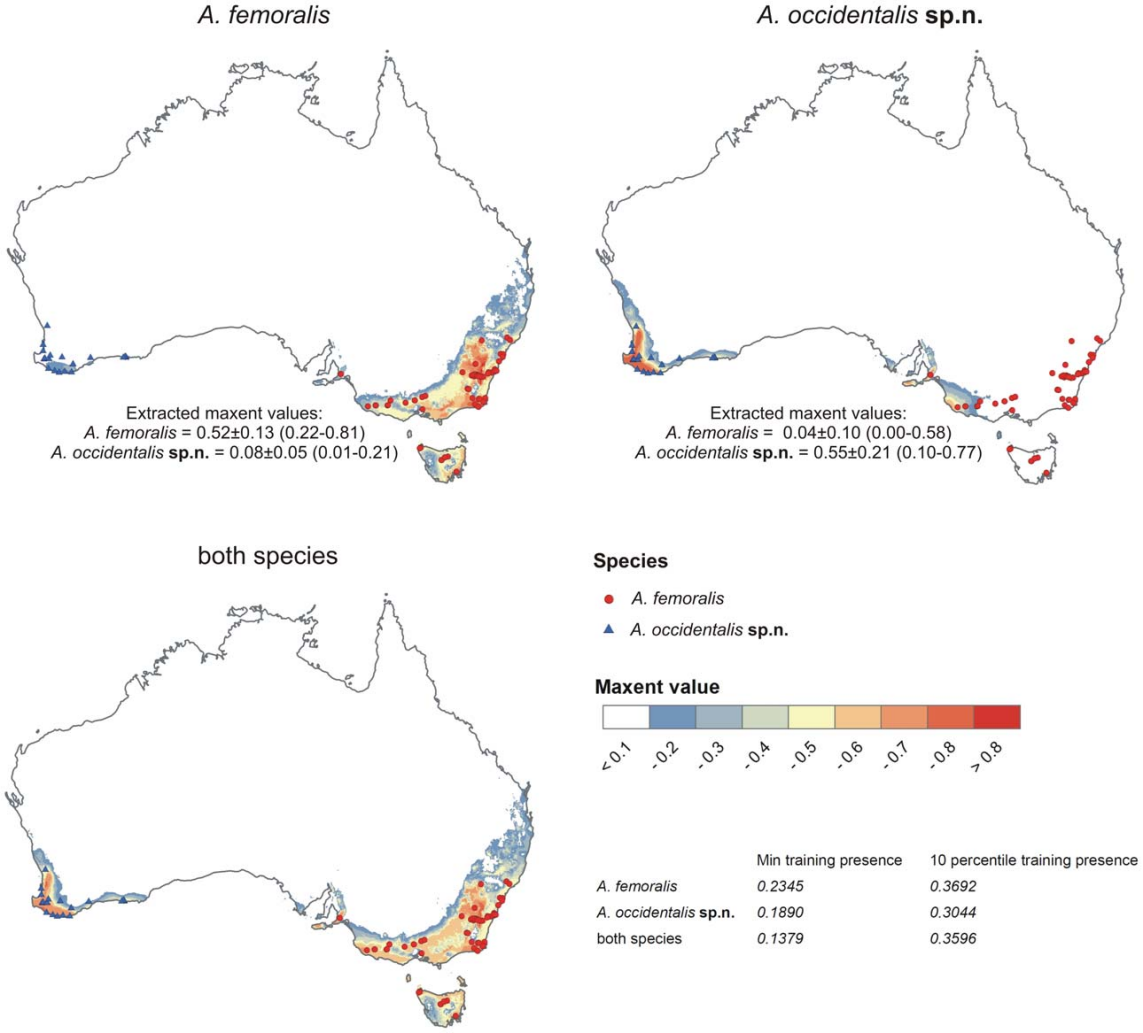
Ecological niche models. Localities of *Antiporus femoralis* (blue triangles) and *Antiporus occidentalis* **sp.n.** (red circles), displayed on the backgrounds of Maxent-created ecological niche models. Higher Maxent values (yellow and red colours) represent areas more suitable for the species according to the Maxent models, lower values (green and blue or white colours) represent areas less suitable.

Ecological niche models are visualised in Fig. 3. According to their AUC values, the ability to distinguish presence from random background points of all models was larger than 0.9 and thus considered ‘very good’ according to the classification of Swets [86]. AUC values were 0.982 for the ENM of eastern and 0.993 for the ENM of western *A. femoralis*. The ENMs of both species together had a slightly lower AUC of 0.977.

Analysis of the environmental variable contribution showed that for the distributions of eastern as well as western *A. femoralis*, “precipitation coldest quarter” was the variable of highest importance (Table S3). “Annual mean temperature” and “annual precipitation” were the second and third most important predictors in the models of eastern *A. femoralis* and of both groups together. “Annual mean temperature” also provided the highest training gain when used in isolation. For western *A. femoralis*, “precipitation warmest quarter” and “precipitation seasonality” were the second and third most important variables. For the model that included both species, variable importance was similar to that found for eastern *A. femoralis*.

The measured niche overlap between eastern and western *A. femoralis* was I = 0.454 and D = 0.192. Values close to 0 describe little overlap in ecological niches and values close to 1 describe high similarity. The overlap between the niches of eastern and western *A. femoralis* can therefore be considered low, judging from these values alone. Note that values of D are generally lower than of I.

**Figure 4:**
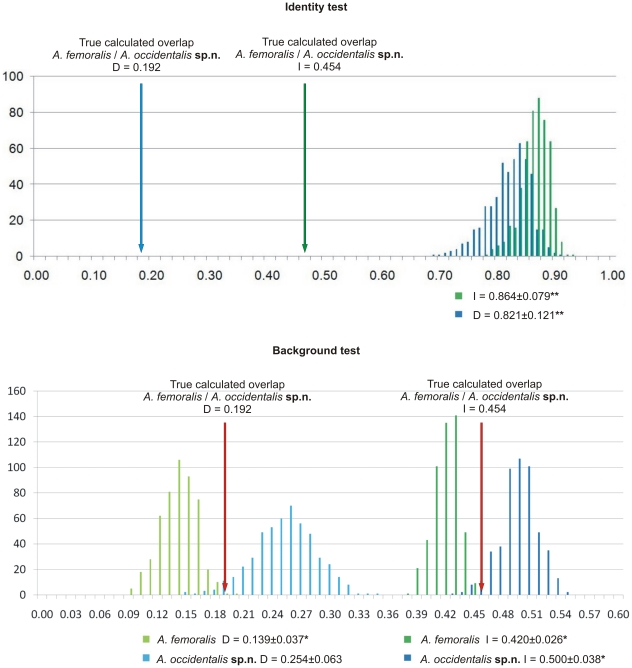
Results of the identity and background tests. Arrows indicate the results of ENMtools' niche overlap test representing the true calculated niche overlap. Columns represent the niche overlap values created in the replicates of the identity and background tests. The true calculated overlap values (I and D) are far outside the 99.9% confidence intervals of the identity test results and thus highly significant (indicated by two asterisks **). For the background tests, results are given for *A. femoralis* (compared to the background of *A. occidentalis* **sp.n.**) and for *A. occidentalis* **sp.n.** (compared to the background of *A. femoralis*). If marked with an asterisk *, the true calculated niche overlaps are outside the 95% confidence intervals but not outside the 99.9% confidence intervals of the background test results and are therefore significant.

The results of the identity and background tests are shown in Fig. 4. According to the identity test, the null hypothesis of niche identity is rejected, meaning that the climate envelopes of eastern and western *A. femoralis*, as modelled here, are highly significantly distinct. In the background test, the null hypothesis that differences in the ecological niches can be explained by environmental differences in their areas of occupancy alone is rejected. The niches are significantly (I and D) more similar than expected based on the distribution of eastern *A. femoralis* and significantly (I only) more different based on the distribution of western *A. femoralis*.

### Taxonomic treatment

Evidence from mtDNA and nDNA sequences, combined with results of ecological niche modelling, suggests presence of two species. *Antiporus femoralis* was described from New South Wales: Sydney, within the geographical range of the eastern clade. Thus, we assign the new species name *A. occidentalis* **sp.n.** to the western clade.

### *Antiporus occidentalis* sp.n

Fig. 5b.

**Figure 5:**
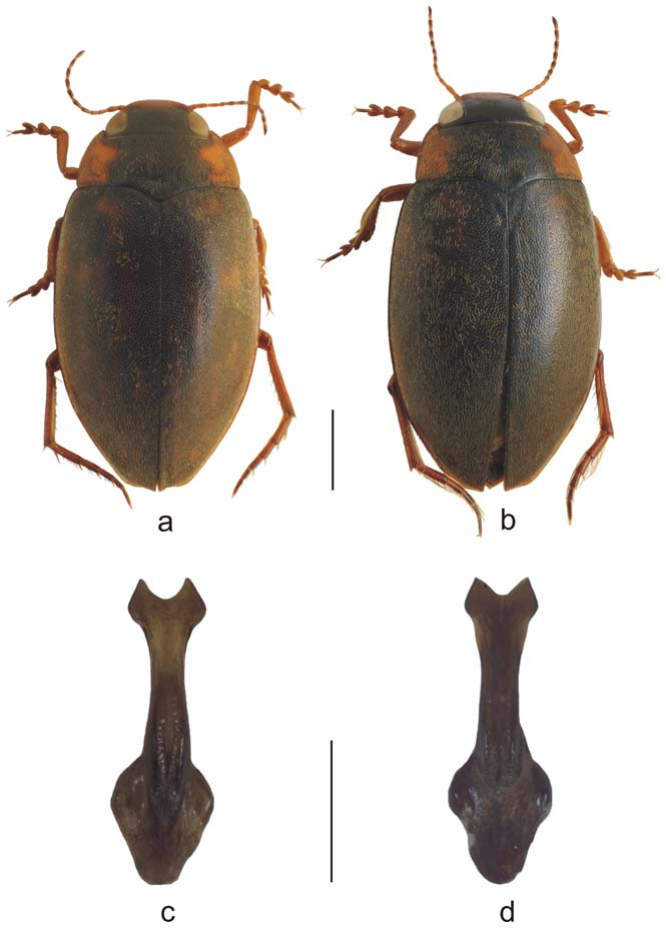
Habitus photographs. Habitus of a) *Antiporus femoralis* (male, SE Australia), b) *A. occidentalis* **sp.n.** (male, SW Australia) (scale bar 1 mm). Ventral views of median lobes of aedeagi of c) *A. femoralis* and d) *A. occidentalis* **sp.n.** (scale bar 0.4 mm). Minor differences between median lobi of *A. femoralis* and *A. occidentalis* **sp.n.** (c, d) are attributed to individual variability. Photos: L. Hendrich.

http://www.species-id.net/w/index.php?title=Antiporus_occidentalis&oldid=2012

urn:lsid:zoobank.org:act:481E4A90-4127-4B33-B878-A35F33A0A35F

#### Type locality

Australia: Western Australia, Lane Poole Conservation Reserve, Nalyerin Lake.

#### Type material

Holotype: Male: “AUSTRALIA/WA: Lane Poole Conservation Reserve, Nalyerin Lake, 300 m, 29. & 30.12.1999, Hendrich leg. (loc.4/151)”, “DNA M.Balke 3757”, [green printed label], “HOLOTYPE *Antiporus occidentalis* **sp.n.** des. 2010” [red label, printed] (WAM).

**Paratypes.** 8 specimens with same locality data as holotype (7 specimens with “DNA M.Balke 3750”, “3751”, “3752”, “3753”, “3754”, “3755”, “3756” [green labels, printed]) and “PARATYPE *Antiporus occidentalis* **sp.n.** Hawlitschek, Hendrich & Balke des. 2010” [red label, printed] (SAMA, CLH, ZSM); 2 exs., “AUSTRALIA, WA, 10 Km S Cataby, Brand Highway, Nammegarra Road, 9.9.2002, 30°53′S 115°36′E, Hendrich leg./Loc. 29/193” (1 specimen with “DNA M. Balke 1421” [green label, printed]) (CLH); 1 ex., “Australia, WA/North of Bunbury, Yalgorup N.P., east of Preston Beach, 0 m, 24.11.1996, L. Hendrich leg./Coll. Lok. 30” (CLH); 3 exs., “Australia,WA/Nannup, “Wildflower Walk” n. Nannup 100 m, 25.11.1996, L. Hendrich leg./Coll. Lok. 32” (CLH); 6 exs., “Australia, WA/Nannup, Balingup-Nannup Road, Revelly Bridge, 130 m, 25.11.1996, L. Hendrich leg./Coll. Lok. 33” (CLH); 1 ex., “Australia, WA/5 km S Northcliffe, 10 m, 27.11.1996, L. Hendrich leg./Coll. Lok. 37” (CLH); 1 ex., “Australia, WA/20 km NW Walpole, Interstate Hwy. No. 1, 27.11.1996, L. Hendrich leg./Coll. Lok. 38” (CLH); 2 exs., “Australia, WA/Walepole-Nornalup N.P., Peaceful Bay, 0 m, 28.11.1996, L. Hendrich leg./Coll. Lok. 39” (CLH); 3 exs., “Australia, WA/Stirling Range N.P., Stirling Range Drive in Richtung Red Gum Pass, 450 m, 29.11.1996, L. Hendrich leg./Coll. Lok. 41” (CLH); 1 ex., “Australia (WA), Nannup envir., roadside creeks, 1.12.95 Pederzani” (CFP); 16 exs., “Australia (WA), Pemberton, pond, Della Franca farm, 3.12.98 Pederzani” (CFP, CSR); 2 exs., “AUSTRALIA/WA: Nannup, Balingup-Nannup Road, Revelly Bridge, 130 m, 31.12.1999, Hendrich leg. (loc.6/153)” (CLH); 3 exs., “AUSTRALIA/WA: 5 km S Northcliffe, 50 m, 2.1.2000, Hendrich leg. (loc.10a/156)” (CLH); 2 exs., “AUSTRALIA/WA: D′Entrecasteaux N.P., 15 km S Northcliffe, Windy Harbour Road, 50 m, 3.1.2000, Hendrich leg. (loc. 10c/156)” (CLH); 1 ex., “AUSTRALIA/WA: Albany Hwy, Muir Lakes Nature Reserve, SW part of Byenup Lagoon, 4.&5.1.2000, Hendrich leg. (loc. 11/157)” (CLH). 1 ex., “WA Cannington 14/08/1924/32°01′00“S 115°57′00“E L. Glauert leg.” [40086] (WAM); 1 ex., “WA Cokatea Creek Tenindewa 8/01/1926” [40708] (WAM); 1 ex., “WA Wanneroo Melaleuca Park, 14/08/1976 31°40′25”S 115°53′23”E Southwell-Keely leg.” [42685] (WAM); 3 exs., “WA Banksiadale 01/05/1969 32°38′S 116°06′E D.S. Adair leg.” [42736, 42737, 42738] (WAM); 3 exs., “WA Bullsbrook Tortoise Reserve 10/1963 31°39′S 115°59′E Zoological Honours Class leg.” [42739, 42740, 42741] (WAM).

#### Etymology

A western Australian species.

#### Description

Body in dorsal view rotundate-oval, convex, widest behind the middle. http://www.species-id.net/o/index.php?title=File:Antiporus_occidentalis_dorsal.jpg&oldid=109760.

#### Measurements

Total length of beetle  =  4.6–4.9 mm (holotype 4.8 mm); total length without head  =  4.4–4.7 mm (holotype 4.6 mm); maximum width  =  2.3–2.5 mm (holotype 2.4 mm).

#### Colour

Upper side reddish brown; some portions with small and less extended dark brown or black patches. Head uniformly black, reddish brown on the anterior part. Antenna testaceous, distal joint apically darkened. Pronotum reddish brown with large patch on middle part which does not reach the anterior border. Elytra reddish brown with small and less extended dark brown or black patches (Fig. 5b). Venter black, including pronotum, epipleuron, metaventrite, metacoxal plate and prosternal process. Legs and abdominal sternites reddish brown.

#### Sculpture

Head finely microreticulated, regularly and densely punctured, coarser around the clypeal grooves. Interstices between punctures larger than the diameter of the punctures, particularly on the disc.

Pronotum semi-matt, very finely microreticulated. Sides of pronotum regularly and gently curved. Puncturation regular on the whole surface, except on a round area situated on both sides of the disc where the punctures are more sparse and on the lateral border where they are coarser and very close. Pronoto-elytral angles obtuse.

Puncturation on elytra regular and very dense, covering the whole surface. The interstices between punctures are narrower than the diameter of punctures, but less so on the apical half. Ground sculpture finely microreticulated, semi-matt on the basal half, shagreened on the apical half.

Ventral surface; prosternal process narrowly lanceolate, rounded tip, weakly carinate in cross section, slightly narrowed between procoxae. Metacoxal lines raised, moderately separated, subparallel in posterior half, diverging to about twice their narrowest width in anterior half. Metacoxae and sternites very strongly punctured.

#### Male

Pro- and mesotarsi moderately expanded, robust; single proclaw thickened, sharply curved and with a small tooth near base. Metafemora slightly incised into a triangular process near apex. Last abdominal sternite rounded in middle. Parameres broad and rounded. Median lobe of aedeagus in ventral view very broad, strongly bilobed towards tip (Fig. 5d), in lateral view rather thin and elongated. Minor differences between median lobi of *A. femoralis* and *A. occidentalis* **sp.n.** (Fig. 5) are attributed to individual variability.

#### Female

Pro- and mesotarsi narrower than in males, not expanded. Proclaws simple. Mesotibia narrow.

#### Affinities

The new species is the sister species of *A. femoralis* and cannot be separated using morphological characters such as size, colour and form of median lobe (Fig. 5). However, the species are allopatric: *Antiporus occidentalis* **sp.n.** occurs in south-western Australia, and *A. femoralis* in south-eastern Australia, south of Brisbane, along the east coast to Victoria, South Australia and Tasmania.

#### Distribution

South-western Australia. South of a line from Carnavon to the Stirling Ranges (Fig. 3).

#### Habitat

*Antiporus occidentalis* **sp.n.** was collected from shaded or at least half-shaded pools, peatland swamps and lakes, overgrown roadside ditches and rest pools of intermittent creeks (Fig. S2), from the coast (Preston Beach near sea level) up to 450 m in the Stirling Ranges. In contrast to the south-eastern Australian *A. femoralis*, it seems that the species prefers more peaty water with a dark bottom consisting of mud, peat and plant debris.

### *Antiporus femoralis* (Boheman, 1858)

Fig. 5a.

*Hydroporus femoralis* Boheman, 1858: 19.

*Antiporus femoralis* (Boheman, 1858): Watts 1978: 67; Brancucci 1984: 151; Watts 1997: 36.

#### Type locality

Australia: New South Wales, Sydney.

#### Material examined

New South Wales: 2 exs., C NSW, 25 km N Wollongong, Darkes Forest, Maddens Fall Lookout, 480 m, 29.X.2006, 34.13.335S 150.54.465E, L. & E. Hendrich leg. (NSW 86); 2 exs., C NSW, 17 km SE Nowra, Jerwis Bay NP, Coonemia Road, 54 m, 31.X.2006, 34.58.156S 150.43.045E, L. & E. Hendrich leg. (NSW 90); 2 exs., C NSW, 1 km N Nowra, Bomaderry, Bomaderry Creek, 71 m, 31.X.2006, 34.50.383S 150.35.509E, L. & E. Hendrich leg. (NSW 91); 3 exs., C NSW, 10 km S Nowra at Falls Creek, Parma Creek, 27 m, 1.XI.2006, 34.58.104S 150.35.415E, L. & E. Hendrich leg. (NSW 92); 2 exs., C NSW, 40 km SW Nowra, Braidwood Road, Tianjara Creek, 498 m, 1.XI.2006, 35.06.382S 150.20.037E, L. & E. Hendrich leg. (NSW 93); 2 exs., C NSW, Endrick River at Braidwood Road, 554 m, 1.XI.2006, 35.05.193S 150.07.182E, L. & E. Hendrich leg. (NSW 94); 8 exs., C NSW, 48 km NE Braidwood, Corang Creek, 589 m, 1.XI.2006, 35.10.488S 150.04.101E, L. & E. Hendrich leg. (NSW 95); 2 exs., C NSW, 10 km W Braidwood, Shoalhaven River at Bombay Bridge, 628 m, 2.XI.2006, 35.25.419S 149.42.582E, L. & E. Hendrich leg. (NSW 96); 10 exs., S NSW, 2 km NE Queanbeyan, Molonglo Gorge, 584 m, 12.XI.2006, 35.19.313S 149.15.029E, L. & E. Hendrich leg. (NSW 101); 2 exs., S NSW, 3 km N Jindabyne, Wollondibby Creek, 928 m, 13.XI.2006, 36.23.406S 148.35.533E, L. & E. Hendrich leg. (NSW 102); 2 exs., S NSW, Mt. Kosciusko NP, Diggers Creek (Alpine lake), 1517 m, 14.XI.2006, 36.21.357S 148.29.281E, L. & E. Hendrich leg. (NSW 106); 4 exs., S NSW, 12 km SW Delegate, Bog Road, 812 m, 15.XI.2006, 37.04.356S 148.53.260E, L. & E. Hendrich leg. (NSW 108); 1 ex., S NSW, Imlay Road, White Rock Picnic Area, 497 m, 15.XI.2006, 37.08.039S 149.21.324E, L. & E. Hendrich leg. (NSW 109); 1 ex., S NSW, Imlay Road, 8.5 km E from Monaro Hwy to Eden, 564 m, 15.XI.2006, 37.08.029S 149.25.028E, L. & E. Hendrich leg. (NSW 110); 2 exs., S NSW, 6.5 km SW Eden, Towamba Road 2 km N Nullica, 556 m, 16.XI.2006, 37.04.412S 149.51.200E, L. & E. Hendrich leg. (NSW 111); 1 ex., S NSW, Wallagaraugh River Picnic Area, 43 km SW Eden, 54 m, 17.XI.2006, 37.22.079S 149.43.073E, L. & E. Hendrich leg. (NSW 112). Victoria: 2 exs., E VIC, Tonghi River at Hwy 1 3–5 km SW Cann River, 126 m, 17.XI.2006, 37.33.503S 149.03.546E, L. & E. Hendrich leg. (VIC 115); 1 ex., S VIC, Simpsons Creek 12 km SW Orbost at Princess Hwy, 31 m, 18.XI.2006, 37.45.095S 149.20.436E, L. & E. Hendrich leg. (VIC 116); 2 exs., C VIC, Hughes Creek at Avenel, 161 m, 25.XI.2006, 36.54.221S 145.14.191E, L. & E. Hendrich leg. (VIC 120); 2 exs., C VIC, 5–7 km W Puckapunyal, street to Tooborac, 205 m, 25.XI.2006, 37.00.282S 144.58.415E, L. & E. Hendrich leg. (VIC 122); 3 exs., C VIC, Kyneton, Boggi Creek, Mineral Springs Picnic Area, 485 m, 26.XI.2006, 37.14.094S 144.25.259E, L. & E. Hendrich leg. (VIC 123); 3 exs., W VIC, Grampians, 7 km NW Dunkeld, street to Lavendish, 229 m, 27.XI.2006, 37.38.510S 142.17.507E, L. & E. Hendrich leg. (VIC 125); 3 exs., W VIC, Grampians, Wannon River, 5 km N Dunkeld, 236 m, 27.XI.2006, 37.37.494S 142.20.226E, L. & E. Hendrich leg. (VIC 126); 2 exs., W VIC, Grampians, Fyans Creek, 15 km S Halls Gap, 363 m, 27.XI.2006, 37.14.595S 142.32.240E, L. & E. Hendrich leg. (VIC 128). South Australia: 1 ex., SE SA, 10–12 km N Mt. Gambier, Mt. Gambier Forest Reserve, 77 m, 29.XI.2006, 37.42.308S 140.47.523E, L. & E. Hendrich leg. (SA 135); 3 exs., SA, Meadows Creek at Kuitpo Forest, 286 m, 3.XII.2006, 35.12.367S 138.42.004E, L. & E. Hendrich leg. (SA 137). Tasmania: 1 ex., NW TAS, Montagu River at Togari, 41 m, 12.XII.2006, 40.54.545S 144.52.399E, L. & E. Hendrich leg. (TAS 145); 3 exs., NW TAS, Welcome River at Hwy A 2, 44 m, 12.XII.2006, 40.57.004S 144.48.325E, L. & E. Hendrich leg. (TAS 146); 10 exs., C TAS, CPCA, 500 m E Lake Ada, pools, 1154 m, 14.XII.2006, 41.52.575S 146.28.432E, L. & E. Hendrich leg. (TAS 149).

#### Description

Morphology and size as in the above species. Minor differences between median lobi of A. *femoralis* and *A. occidentalis* **sp.n.** (Fig. 5) are attributed to individual variability.

Remarks. Specimens from Tasmania are larger and darker than specimens from the mainland.

#### Distribution

South-eastern Australia. From around Sydney along the east coast south to Victoria, Tasmania and South Australia, including Port Lincoln and Kangaroo Island (Watts 1978, 1997) (Fig. 3).

#### Habitat

The species inhabits a wide variety of freshwater habitats and can be found in slow-flowing creeks, rest pools of intermittent streams and rivers, ponds, old farm dams, ditches, and seasonal or permanent sedge swamps from near sea level up to an altitude of 1154 m. The ideal habitat should be rich in rotten leaves or plant debris and overgrown with sedges or reed (Fig. S2).

## Discussion

### Taxonomy

We strongly support the utilisation of Internet technology to enhance dissemination of taxonomic knowledge (e.g., Knapp [93] for an example from this journal), like SpeciesID (http://www.species-id.net/w/index.php?title=Antiporus_occidentalis&oldid=2012). WikiSpecies pages, in our opinion the best taxonomic information facility on the web (see also Page [94] on WikiPedia), are given in Text S1. The species pages have links to GenBank entries and additional material such as habitat photos. Where necessary, they will be updated to provide further data as they become available.

### Phylogeny

We inferred a fully resolved phylogeny for 10 of the 16 species of the genus *Antiporus*, with four major lineages. *Antiporus gottwaldi* is the sister taxon to all other species. Next, *A. hollingsworthi* is the sister taxon to all remaining species except *A. gottwaldi*. The remaining species are divided into two clades. Species from the clade including *A. femoralis* are distributed in the southern parts of Australia (from southern New South Wales, Victoria, Tasmania, South Australia and the southern part of Western Australia), while members of the clade including *A. bakewellii* range from Northern Territory to northern Queensland (*A. jenniferae*) and down the east coast to Tasmania (*A. blakeii*). Although *A. blakeii* and *A. femoralis* belong to separate clades, their distribution is almost congruent. These broadly sympatric species belong to different clades. Occasionally, both species can be found in the same habitat (e.g., Tasmania, Victoria).

### Ecological niche modelling of *A. femoralis* and *A. occidentalis* sp.n

**Figure 6:**
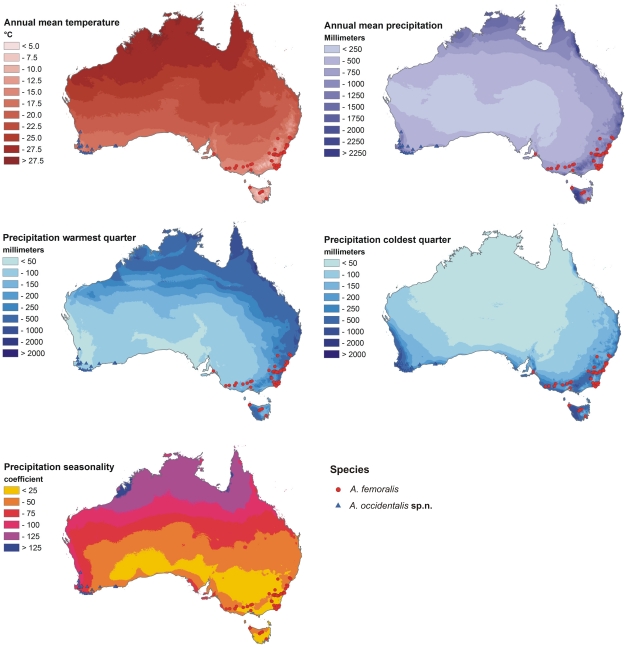
Climate variables. A projection of *Antiporus femoralis* (blue triangles) and *Antiporus occidentalis* **sp.n.** (red circles) localities on climate variables. Note that localities of both taxa are situated in areas with relatively high precipitation in the coldest quarter. In the warmest quarter, most localities of *A. femoralis* also receive high precipitation, while localities of *A. occidentalis* **sp.n.** are predominantly dry in this season. This effect is also visualised as precipitation seasonality, where *A. femoralis* inhabit areas with relatively low precipitation seasonality, and *A occidentalis* **sp.n.** inhabit areas with moderate precipitation seasonality.

Our results indicate that *A. femoralis* and *A. occidentalis* **sp.n.** differ in their realised ecological niches, represented here by their modelled climate envelopes. As suggested by the results shown in Fig. 6, the distributions of both species depend heavily on winter rain. However, the variables representing a high level of seasonal variation in climate (“precipitation warmest quarter” and “precipitation seasonality”) are more important in the *A. occidentalis* **sp.n.** model, while in the *A. femoralis* model they are of lower relative importance than “annual mean temperature” and “annual precipitation”.

As shown in Fig. 3, the distributions both of *A. femoralis* and *A. occidentalis* **sp.n.** cover areas highly suitable for both taxa according to the ENMs. However, *A. femoralis* is commonly found at localities that are less suitable for *A. occidentalis* **sp.n.** and vice versa.

Fig. 5 shows that areas of occupancy of both *A. femoralis* and *A. occidentalis* **sp.n.** correspond to relatively high precipitation in the Southern Hemisphere winter (coldest quarter). However, *A. femoralis* lives in areas where summer (warmest quarter) precipitation is at a level similar to that in winter, whereas *A. occidentalis* **sp.n.** inhabits areas with very dry summers. Apparently, the main difference between the climatic envelopes of these two *Antiporus* species is the summer drought in the area of *A. occidentalis* **sp.n.** The ecological validity of this difference is also confirmed by the decrease in regularized training gain if “precipitation warmest quarter” is omitted from the model of *A. occidentalis* **sp.n.** This pattern suggests a possible niche divergence between the two taxa, with *A. occidentalis* **sp.n.** showing a preference for areas with lower summer precipitation and *A. femoralis* preferring areas with relatively wet summers and low seasonal differences in precipitation. Given the nature of ENMs, especially the restricted set of abiotic variables and the complete exclusion of biotic variables from the analyses, such results must be treated with caution [33], [80]. The apparent divergence in climatic envelopes might be due to abiotic factors not included in the analysis, such as differences in microhabitat structures, soil or water chemistry. It might also be influenced by biotic factors. Thise might, for example, be predators or competing species present in the area of *A. occidentalis* **sp.n.** Both species occur in syntopy with several other species of dytiscid beetles with similar ecology, but none of these syntopic similar species are present in the ranges of both *Antiporus* species [53]. The presence of these other species might keep *A. occidentalis* **sp.n.** from occupying the niches of its Western sibling taxon, *A. femoralis*.

We used two approaches to model validation to address these possible problems. First, the hypothesis that *A. femoralis* and *A. occidentalis* **sp.n.** occupy different environmental niches was tested by comparing ENMs of the two species to a model based on both species together. As described in Raxworthy [24], species delimitation by ecological niche modelling is most reliable if models of each split clade alone are superior (according to better fit and more significant statistical model validation) to models of all clades lumped together. Models in which this is not the case might also be validated by including negative locality data (but see [95]), which in the present case has not been available. As all models have fits considered “very good”, this criterion does not contribute to the verification of species delimitation in the case of *A. occidentalis* **sp.n.**

Another method of model validation is the use of various statistical tests, as implemented in the ENMtools software [88], [90]. According to the results of the identity test, niche diversification between these two sibling species must be considered highly significant. The climate envelope of *A. occidentalis* **sp.n.** is very different from that of *A. femoralis*. The background test yields results in which the significance is much smaller in magnitude than that of the identity test results. Nevertheless, the background test results indicate that this divergence cannot be attributed to the ecological difference in the species' allopatric ranges alone. This suggests that in the area of occupancy of *A. occidentalis* **sp.n.**, a different climate space is available than in the range of *A. femoralis*.

However, the results of the two test runs seem to contradict each other. The climate envelopes of both species are more divergent than expected based on localities of *A. occidentalis* **sp.n.** (and on random test samples drawn from the background of *A. femoralis*), but they are more similar than expected based on the reverse comparison (Fig. 4, explained in Fig. S3). Nakazato et al. [96] performed background tests for species distribution models of four sibling species pairs and obtained a variety of outcomes. Whereas identity tests yielded highly significant results, sibling species were either ecologically more divergent, less divergent or not significantly divergent according to the background tests. One case resembled that of *Antiporus*: species were either more or less divergent depending on the direction of the test. The authors explain this counterintuitive result by differences in the heterogeneity of the species' environmental backgrounds.

In our view, the identity tests clearly indicates that *A. femoralis* and *A. occidentalis* **sp.n.** are ecologically divergent. This divergence may result from their exposure to different environmental backgrounds alone, but it may also be result from evolutionary niche diversification. The results of the background test do not contradict the latter assumption. They simply state that this diversification is higher than expected if tested one way and lower than expected if tested the other way.

### Speciation/species delimitation in *A. femoralis* and *A. occidentalis* sp.n

In our view, *A. occidentalis* **sp.n.** constitutes a valid species according to the unified species concept, as it represents a metapopulation lineage evolving separately from other metapopulation lineages, including that represented by its closest relative, *A. femoralis*. In this paper, we used two different approaches to validate this hypothesis. First, a taxonomic/phylogenetic approach using morphological and molecular genetic data was employed. The morphological analysis showed that *A. occidentalis* **sp.n.** is indistinguishable from *A. femoralis*. Genetic data, however, unambiguously supported presence of two clades, and the relatively high cox1 divergence (>6%) clearly suggested further investigation into the possible presence of a cryptic species [97]. The operational criterion applicable to this result is the genotypic cluster of Mallet [19]. This criterion defines species as identifiable clusters having no intermediates.

In our second approach, we used ecological data to test the ecological species concept, as proposed in Van Valen [17] and Andersson [18], as operational criterion. According to this concept, individuals occupying the same niche or adaptive zone constitute a species. The results of our modelling suggest that *A. femoralis* and *A. occidentalis* **sp.n.** do not occupy the same niche. The difference in their niches can be attributed largely, but not completely to the different environmental conditions prevailing in their distributional ranges. The distributional range of *A. occidentalis* **sp.n.** features drier summers and generally higher seasonal variation in precipitation than those experienced by *A. femoralis*. In our view, these two operational criteria support the assessment of *A. occidentalis* **sp.n.** as a separately evolving metapopulation lineage.

Precise estimation of the age of separation using a molecular clock approach is difficult due to the lack of reliable calibration points. Other pairs of dytiscid species (*Hyderodes shuckardi* Hope, 1838 and *H. crassus* Sharp, 1882, *Spencerhydrus latecinctus* Sharp, 1882 and *S. pulchellus* Sharp, 1882) are known to exhibit a distribution pattern similar to *A. femoralis* and *A occidentalis* **sp.n.**, but no studies on molecular dating have yet been performed. The observed intraspecific distance suggests that *A. femoralis* and *A. occidentalis* **sp.n.** have remained in evolutionary separation for a long time. Applying the “molecular clock” evolution rates of about 3.54% divergence per million years (myr) of Papadopoulou et al. [98] to the minimum interspecific cytochrome c oxidase 1 distance suggests that the two lines have split around 1.0 to 1.9 myr ago. As shown by various studies, age estimations using standard mutation rates must be viewed with great caution [99]–[102]. Nevertheless, this result supports the view that speciation between *A. femoralis* and *A. occidentalis* **sp.n.** took place well after the Miocene transgression period, when the Nullarbor plain had already fallen dry. In this scenario, speciation probably followed a colonization event across the arid plain, possibly during a temporary phase of less arid conditions.

The scenario presented here attempts to connect present biodiversity with evidence from the geological record. It is based on several assumptions, for some of which evidence is scarce, but offers one possible explanation for the two morphologically indistinguishable, but genetically and ecologically divergent sibling species *A. femoralis* and *A. occidentalis* **sp.n.** It may be supported by future studies on similar speciation events, especially if more accurate age estimations are possible. We believe that the results of such studies may help elucidate the implications of geological history and past environmental changes for Australia's present biogeography.

## Supporting Information

Table S1 **Sequences of primers used for PCR and sequencing.** Forward (F) and reverse (R) primers are given. Mitochrondrial gene loci: coi  =  cytochrome C oxidase 1, cob  =  cytochrome B oxidase, 16S  =  16S ribosomal RNA. Nuclear gene loci: H3  =  histone 3, 18S  =  18S ribosomal RNA, ArK  =  arginine kinase.
(DOC)

Table S2 **Coordinates of** ***Antiporus femoralis*** **and** ***A. occidentalis*** **used for modeling.** Geographic latitude and longitude are given in decimal degrees.
(DOC)

Table S3 **A heuristic estimate of the contributions of the bioclimatic variables used for modelling.** Results of the jackknife analysis of variable importance are given as ranks (1 to 5) for all variables. Isolation: rank of the variable's training gain when used in isolation. Omission: rank of the variable in decreasing the total regularised training gain when omitted.
(DOC)

Figure S1 **Background selection in ecological niche modelling.** This picture shows each two ecological niche models for *Antiporus femoralis*, *A. occidentalis* **sp.n.** and both species together. For each set of locality data, one model was created using a manually specified background, as indicated by the green frame, and another one using no specified background. Both models were tested for niche overlap. All resulting values of I and D are close to 1 and thus indicate high overlap between models, confirming the similarity apparent from visual comparison.
(TIF)

Figure S2 **Habitat of** ***Antiporus occidentalis*** **sp.n.** a) Pond near Preston Beach, Western Australia (Loc. 30) and b) seasonal swamp at “Nannup Wildflower Walk” near Nannup, Western Australia (Loc. 32,).
(TIF)

Figure S3 **Apparent contradiction in the background test results.** This picture (modified from Nakazato et al. [96]) shows the environmental spaces available to (red and blue lines) and occupied by (shaded areas) both allopatric *Antiporus* species. In the niche overlap test, true localities of both species are compared. In the background test, the true localities of each one species are compared to random samples points drawn from the background areas (i.e., available environmental spaces) of the other species. Here, background test (1) yields relatively more divergent results than the true calculated overlap because, although the same overlap exists, it includes much more non-overlapping environmental space. Background test (2) yields more similar results than the true calculated overlap because it includes far more overlap than non-overlap between niche spaces. See Fig. 3.
(TIF)

Text S1 **Web links.** *Antiporus femoralis* and *A. occidentalis* **sp.n.** on Wikispecies.
(DOC)
