# Supplementary material for: Ecological Niche Modelling and nDNA Sequencing Support a New, Morphologically Cryptic Beetle Species Unveiled by DNA Barcoding
Source: PLoS One. 2011 Feb 9;6(2):e16662. doi: 10.1371/journal.pone.0016662 (PMC3036709; doi:10.1371/journal.pone.0016662)
Supplement: Table S1 — Sequences of primers used for PCR and sequencing. Forward (F) and reverse (R) primers are given. Mitochrondrial gene loci: coi = cytochrome C oxidase 1, cob = cytochrome B oxidase, 16S = 16S ribosomal RNA. Nuclear gene loci: H3 = histone 3, 18S = 18S ribosomal RNA, ArK = arginine kinase. (DOC) [file pone.0016662.s001.doc]

**Table S1: Sequences of primers used for PCR and sequencing.**

Forward (F) and reverse (R) primers are given. Mitochrondrial gene loci: coi = cytochrome C oxidase 1, cob = cytochrome B oxidase, 16S = 16S ribosomal RNA. Nuclear gene loci: H3 = histone 3, 18S = 18S ribosomal RNA, ArK = arginine kinase.

| **Gene** | **Primer** | **F/R** | **Primer sequence** | **Reference** |
| --- | --- | --- | --- | --- |
| CO1 | Jerry | F | CAA CAT TTA TTT TGA TTT TTT GG | [Error: Reference source not found] |
|  | Pat | R | TCC AAT GCA CTA ATC TGC CAT ATT A |  |
| CytB | CB3 | F | GAG GAG CAA CTG TAA TTA CTA A | [Error: Reference source not found] |
|  | CB4 | R | AAA AGA AA(AG) TAT CAT TCA GGT TGA AT |  |
| 16S | M14 | F | CGC CTG TTT AAC AAA AAC AT | [Error: Reference source not found] |
|  | M223 | R | GGT CCC TTA CGA ATT TGA ATA TAT CCT |  |
| H3 | H3aF | F | ATG GCT CGT ACC AAG CAG AC(AG) CGC | [Error: Reference source not found] |
|  | H3aR | R | ATA TCC TT(AG) GGC AT(AG) AT(AG) GTG AC |  |
| 18S | 18S 5' | F | GAC AAC CTG GTT GAT CCT GCC AGT (1) | [Error: Reference source not found] |
|  | 18S b5.0 | R | TAA CCG CAA CAA CTT TAA T (1) |  |
| ArK | AK183F | F | GATTCTGGAGTCGGNATYTAYGCNCCYGAYGC | [Error: Reference source not found] |
|  | AK939R | R | GCCNCCYTCRGCYTCRGTGTGYTC |  |

1. Simon C, Frati F, Beckenbach AT, Crespi B, Liu H, et al. (1994) Evolution, weighting, and phylogenetic utility of mitochondrial gene sequences and a compilation of conserved polymerase chain reaction primers. Ann Entomol Soc Am 87: 651-701.
2. Baraclough TG, Hogan JE, Vogler AP (1999) Testing whether ecological factors promote cladogenesis in a group of tiger beetles (Coleoptera: Cicindelidae). Proc. R. Soc. Lond. B 266: 1061-1067.
3. Colgan DJ, McLauchlan A, Wilson GDF, Livingston SP, Edgecombe GD, et al. (1998) Histone H3 and U2 snRNA DNA sequences and arthropod molecular evolution. Aust J Zool 46: 419-437.
4. Shull VL, Vogler AP, Baker MD, Maddison DR, Hammond PM (2001) Sequence Alignment of 18S Ribosomal RNA and the Basal Relationships of Adephagan Beetles: Evidence for Monophyly of Aquatic Families and the Placement of Trachypachidae. Syst Biol 50: 945-969.
5. Wild AL, Maddison DR (2008) Evaluating nuclear protein-coding genes for phylogenetic utility in beetles. Mol Phylogenet Evol 48: 877-891.
