# Supplementary material for: Ecological Niche Modelling and nDNA Sequencing Support a New, Morphologically Cryptic Beetle Species Unveiled by DNA Barcoding
Source: PLoS One. 2011 Feb 9;6(2):e16662. doi: 10.1371/journal.pone.0016662 (PMC3036709; doi:10.1371/journal.pone.0016662)
Supplement: Table S2 — Coordinates of Antiporus femoralis and A. occidentalis used for modeling. Geographic latitude and longitude are given in decimal degrees. (DOC) [file pone.0016662.s002.doc]

**Table S2: Coordinates of *Antiporus femoralis* and *A. occidentalis* used for modeling.**

Geographic latitude and longitude are given in decimal degrees.

| *Antiporus femoralis* | | *A. femoralis* (continued) | | *Antiporus occidentalis* **sp.n.** | |
| --- | --- | --- | --- | --- | --- |
| Latitude | Longitude | Latitude | Longitude | Latitude | Longitude |
| -35.2667 | 149.1000 | -32.6000 | 149.5833 | -31.4113 | 116.0069 |
| -35.3000 | 149.1333 | -35.0833 | 148.1000 | -32.8731 | 115.6802 |
| -35.2500 | 149.1667 | -33.8833 | 151.2167 | -34.0053 | 115.7387 |
| -35.3167 | 148.8000 | -35.4333 | 149.5333 | -33.9201 | 115.8057 |
| -35.4000 | 149.0167 | -35.4000 | 149.3833 | -34.7392 | 116.0864 |
| -35.2500 | 148.9500 | -32.5667 | 151.7833 | -34.9242 | 116.5693 |
| -35.2500 | 149.1167 | -37.6333 | 141.3833 | -35.0380 | 116.9231 |
| -36.3560 | 148.4880 | -37.6333 | 141.3833 | -34.3833 | 117.9833 |
| -37.1398 | 149.3554 | -37.6333 | 141.3833 | -33.8333 | 122.1000 |
| -34.9684 | 150.5903 | -35.2061 | 138.7067 | -33.7833 | 122.0167 |
| -35.1748 | 150.0684 | -37.7051 | 140.7921 | -33.9667 | 116.1333 |
| -35.4237 | 149.7097 | -41.8763 | 146.4739 | -34.9667 | 117.3500 |
| -35.3219 | 149.2548 | -40.9091 | 144.8733 | -33.8833 | 119.3333 |
| -37.1382 | 149.4213 | -41.8763 | 146.4739 | -35.0000 | 117.8667 |
| -35.1064 | 150.3395 | -40.9567 | 144.8054 | -33.8333 | 117.1500 |
| -36.3901 | 148.5922 | -41.8763 | 146.4739 | -33.3500 | 115.6833 |
| -37.0735 | 149.8533 | -42.7833 | 147.6333 | -34.3833 | 118.0000 |
| -35.0866 | 150.1197 | -41.6000 | 146.9667 | -33.8500 | 121.8833 |
| -35.3219 | 149.2548 | -41.6833 | 146.7167 | -33.7000 | 116.2333 |
| -34.2223 | 150.9078 | -37.2490 | 144.4210 |  |  |
| -34.8397 | 150.5918 | -37.2433 | 142.5373 |  |  |
| -34.9684 | 150.5903 | -37.6418 | 142.2918 |  |  |
| -37.0726 | 148.8877 | -37.2490 | 144.4210 |  |  |
| -37.3798 | 149.7288 | -37.6249 | 142.3371 |  |  |
| -34.9693 | 150.7242 | -37.0047 | 144.9736 |  |  |
| -35.1748 | 150.0684 | -37.5584 | 149.0591 |  |  |
| -32.3833 | 151.5500 | -36.9037 | 145.2365 |  |  |
| -33.6500 | 151.0500 | -37.7658 | 149.3406 |  |  |
| -33.6833 | 151.2667 | -37.4333 | 143.7000 |  |  |
| -34.4333 | 149.1000 | -37.9500 | 145.0000 |  |  |
| -35.3333 | 149.3667 |  |  |  |  |
