# Supplementary material for: Ecological Niche Modelling and nDNA Sequencing Support a New, Morphologically Cryptic Beetle Species Unveiled by DNA Barcoding
Source: PLoS One. 2011 Feb 9;6(2):e16662. doi: 10.1371/journal.pone.0016662 (PMC3036709; doi:10.1371/journal.pone.0016662)
Supplement: Table S3 — A heuristic estimate of the contributions of the bioclimatic variables used for modelling. Results of the jackknife analysis of variable importance are given as ranks (1 to 5) for all variables. Isolation: rank of the variable's training gain when used in isolation. Omission: rank of the variable in decreasing the total regularised training gain when omitted. (DOC) [file pone.0016662.s003.doc]

**Table S3: A heuristic estimate of the contributions of the bioclimatic variables used for modelling.**

Results of the jackknife analysis of variable importance are given as ranks (1 to 5) for all variables. Isolation: rank of the variable's training gain when used in isolation. Omission: rank of the variable in decreasing the total regularised training gain when omitted.

| ***A. femoralis*** | | | | |
| --- | --- | --- | --- | --- |
| **Variable** | **Definition** | **% contribution** | **isolation** | **omission** |
| BIO19 | Precipitation coldest quarter | 40.4 | 2 | 4 |
| BIO1 | Annual mean temperature | 27.5 | **1** | 3 |
| BIO12 | Annual precipitation | 18.0 | 3 | 2 |
| BIO18 | Precipitation warmest quarter | 9.4 | 5 | 5 |
| BIO15 | Precipitation seasonality | 4.7 | 4 | **1** |
| ***A. occidentalis* sp.n.** | | | | |
| **Variable** | **Definition** | **% contribution** | **isolation** | **omission** |
| BIO19 | Precipitation coldest quarter | 55.9 | **1** | 2 |
| BIO18 | Precipitation warmest quarter | 23.1 | 3 | **1** |
| BIO15 | Precipitation seasonality | 12.3 | 5 | 3 |
| BIO1 | Annual mean temperature | 7.6 | 2 | 5 |
| BIO12 | Annual precipitation | 1.1 | 4 | 4 |
| **Both species** | | | | |
| **Variable** | **Definition** | **% contribution** | **isolation** | **omission** |
| BIO19 | Precipitation coldest quarter | 54.1 | 2 | **1** |
| BIO1 | Annual mean temperature | 26.1 | **1** | 4 |
| BIO12 | Annual precipitation | 13.4 | 3 | 2 |
| BIO18 | Precipitation warmest quarter | 4.3 | 5 | 5 |
| BIO15 | Precipitation seasonality | 2.1 | 4 | 3 |
