# Supplementary figures and images for: Ecological Niche Modelling and nDNA Sequencing Support a New, Morphologically Cryptic Beetle Species Unveiled by DNA Barcoding
Source: PLoS One. 2011 Feb 9;6(2):e16662. doi: 10.1371/journal.pone.0016662 (PMC3036709; doi:10.1371/journal.pone.0016662)

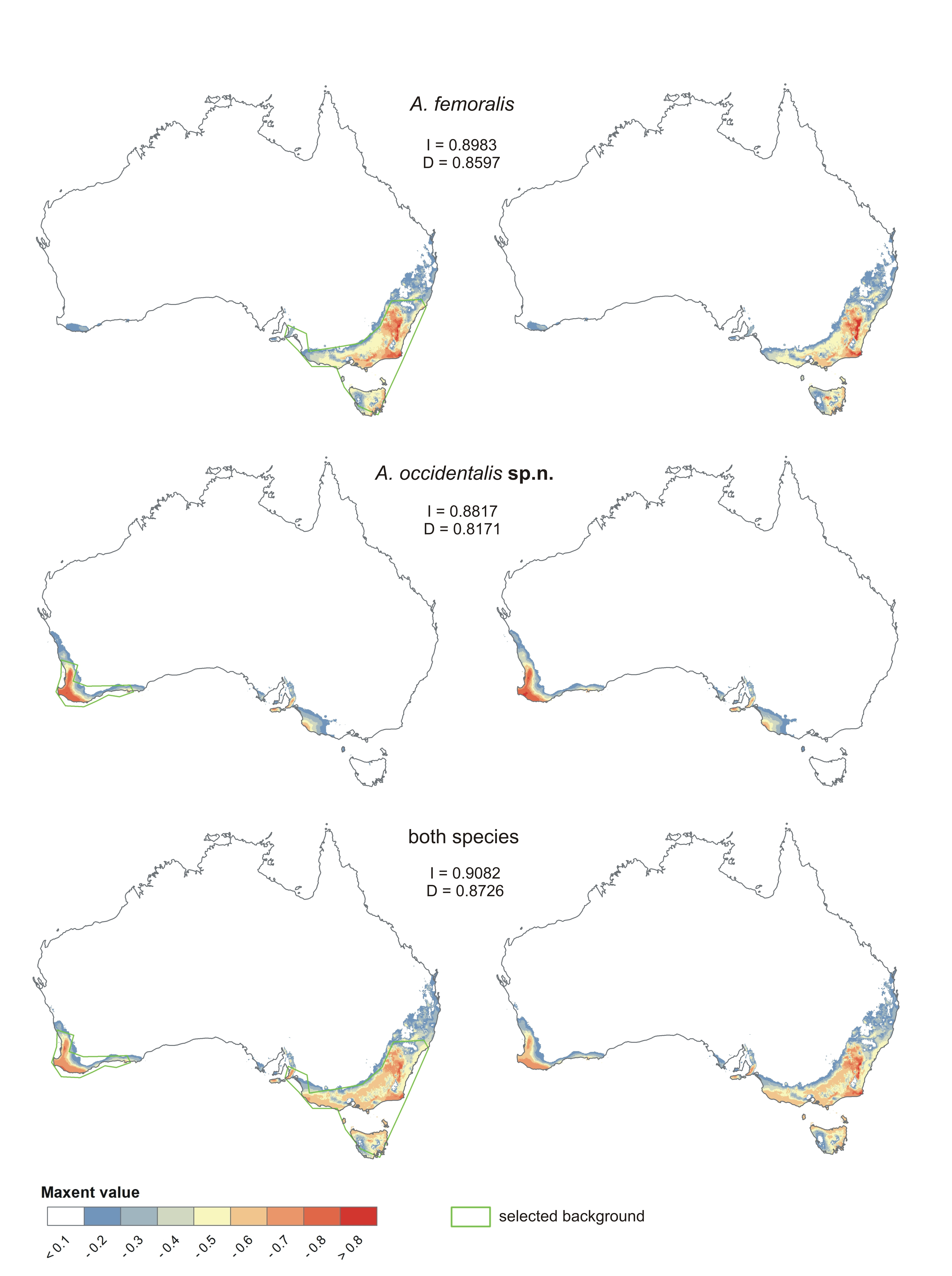

Supplement: Figure S1 — Background selection in ecological niche modelling. This picture shows each two ecological niche models for Antiporus femoralis, A. occidentalis sp.n. and both species together. For each set of locality data, one model was created using a manually specified background, as indicated by the green frame, and another one using no specified background. Both models were tested for niche overlap. All resulting values of I and D are close to 1 and thus indicate high overlap between models, confirming the similarity apparent from visual comparison. (TIF) [file pone.0016662.s004.tif]

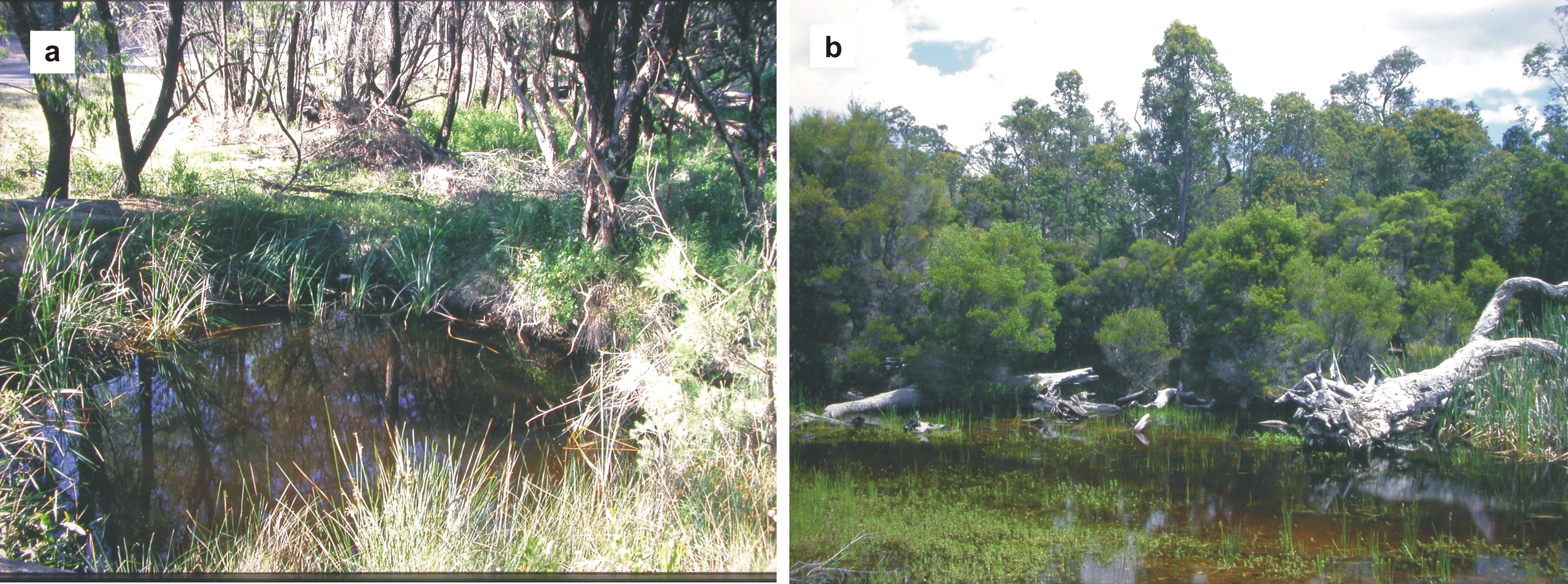

Supplement: Figure S2 — Habitat of Antiporus occidentalis sp.n. a) Pond near Preston Beach, Western Australia (Loc. 30) and b) seasonal swamp at “Nannup Wildflower Walk” near Nannup, Western Australia (Loc. 32,). (TIF) [file pone.0016662.s005.tif]

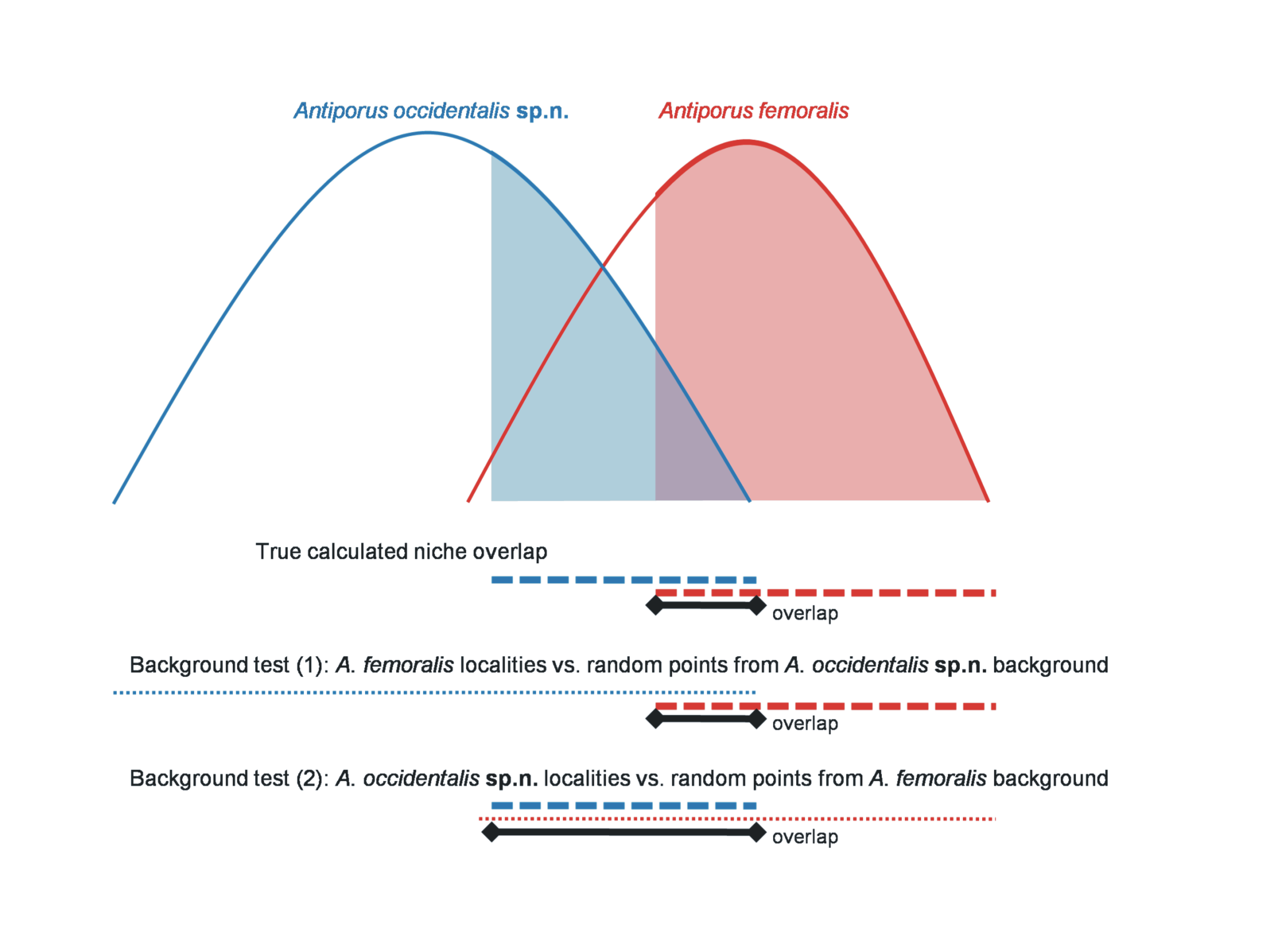

Supplement: Figure S3 — Apparent contradiction in the background test results. This picture (modified from Nakazato et al. [96]) shows the environmental spaces available to (red and blue lines) and occupied by (shaded areas) both allopatric Antiporus species. In the niche overlap test, true localities of both species are compared. In the background test, the true localities of each one species are compared to random samples points drawn from the background areas (i.e., available environmental spaces) of the other species. Here, background test (1) yields relatively more divergent results than the true calculated overlap because, although the same overlap exists, it includes much more non-overlapping environmental space. Background test (2) yields more similar results than the true calculated overlap because it includes far more overlap than non-overlap between niche spaces. See Fig. 3. (TIF) [file pone.0016662.s006.tif]
