# Supplementary material for: Ecological Niche Modelling and nDNA Sequencing Support a New, Morphologically Cryptic Beetle Species Unveiled by DNA Barcoding
Source: PLoS One. 2011 Feb 9;6(2):e16662. doi: 10.1371/journal.pone.0016662 (PMC3036709; doi:10.1371/journal.pone.0016662)
Supplement: Text S1 — Web links. Antiporus femoralis and A. occidentalis sp.n. on Wikispecies. (DOC) [file pone.0016662.s007.doc]

**Text S1: Web links.**

*Antiporus femoralis* and *A. occidentalis* **sp.n.** on Wikispecies.

<http://species.wikimedia.org/wiki/Antiporus_femoralis>

Habitus photographs:

<http://commons.wikimedia.org/wiki/File:Antiporus_occidentalis_dorsal.jpg>

Habitat photographs:

[http://farm5.static.flickr.com/4138/4852983412_eebca8dee3_b.jpg](http://service.gmx.net/de/cgi/derefer?TYPE=3&DEST=http%3A%2F%2Ffarm5.static.flickr.com%2F4138%2F4852983412_eebca8dee3_b.jpg)

[http://farm5.static.flickr.com/4142/4852983786_bd0eb33967_b.jpg](http://service.gmx.net/de/cgi/derefer?TYPE=3&DEST=http%3A%2F%2Ffarm5.static.flickr.com%2F4142%2F4852983786_bd0eb33967_b.jpg)
